# Social capital and oral health promotion: Past, present, and future challenges

**DOI:** 10.3389/froh.2022.1075576

**Published:** 2022-11-25

**Authors:** Jessica Klöckner Knorst, Mario Vianna Vettore, Thiago Machado Ardenghi

**Affiliations:** ^1^ Department of Stomatology, School of Dentistry, Universidade Federal de Santa Maria, Santa Maria, Brazil; ^2^ Department of Health and Nursing Sciences, University of Agder, Kristiansand, Norway

**Keywords:** health promotion, oral health, social capital, social networks, social support

## Abstract

Social capital has been widely inserted in health discussions in recent decades. In this sense, social capital has become a popular term and has been highlighted as one of the main determinants of health in the conceptual framework of the social determinants of the World Health Organization. The concept of social capital focuses on the positive consequences of sociability and places these consequences in the broader discussion of capital. In this sense, social capital reflects the benefits that individuals and communities derive from having broad social networks or high levels of social trust. Despite controversies regarding its definition and numerous criticisms, a growing body of evidence suggests that high levels of social capital benefit oral health. This factor has also been recognized as a potential softener of the impact of oral conditions on oral health, through behavioural and psychosocial processes. Thus, efforts to reduce inequities in oral health preferably should be based on their origins and on their complex causal process, such as the social determinants. The future challenges in the area are specially related to the development of interventions and health promotion actions that aim to stimulate social capital, aiming to reduce the impact of social inequalities on oral health throughout the life course.

## Background

It is increasingly recognized that oral diseases are determined by several biological, psychological, behavioural, social, environmental, and political factors. In this context, a growing body of evidence has explored broader factors that influence oral health outcomes more distally. Among these factors is social capital, which has been highlighted as one of the main social determinants of health. Despite controversies regarding its definition and numerous criticisms regarding its dark side, previous literature has suggested the positive influence of social capital on health outcomes. In our perspective, the concept of social capital focuses on the positive consequences of sociability and places these consequences in the broader discussion of capital. Thus, social capital can be defined as social resources accessed by individuals with good social networks or living in socially structured communities, which generate returns and benefits for themselves or the whole. Notwithstanding, to understand the divergences in the literature and the existing gaps, a deep review of the historical and theoretical aspects surrounding the concept remained necessary, as well as the current contribution of social capital research in health outcomes.

## A historical introduction: Reviewing concepts and definitions

Social capital came into evidence in scientific research, mainly by Pierre Bordieu (1986), James Coleman (1988) and Robert Putnam (1993); however, there is still no consensus on its definition (1–3). For some authors, social capital has been described as the characteristics of the social structure (such as civic participation, levels of trust and reciprocity) that act as resources accessed by individuals and that may facilitate collective actions (2, 3). Social capital has also been defined as a resource accessible to individuals through participation in diverse types of social networks, enabling the achievement of certain goals, returns, or benefits that not be accomplished in the absence of this “characteristic capital” (2–4).

In recent decades, the concepts and applications of social capital have spread to different research areas. However, its theoretical development and this migration from sociology to other disciplines contributed to the divergence of concepts and several classifications of social capital. Some researchers have suggested that social capital has two dimensions: structural and cognitive (5, 6); the structural component is the quantitative side of social capital, which refers to the extent and intensity of participation in associations or other forms of social activity; and the cognitive component is the qualitative side, referring to people's perception of interpersonal trust, solidarity, and reciprocity (5, 6). The perceived trust may also be divided into thick (deep and lasting ties) and thin (superficial and infrequent ties) (7).

Another relevant definition related to social capital are the distinction among bonding, bridging and linking of social capital (8). The “bonding” social capital refers to relationships among members from a network of similar individuals, such as the connection with family members and friends. The “bridging” social capital is related to the ties that connected broader networks, such as individuals from different communities. These two concepts have also been defined as egalitarian or “horizontal” relationships. Finally, “linking” social capital refers to the relationships among individuals or groups in different positions of influence in society, which also is defined as hierarchical or “vertical” relationships (8).

Whether social capital is an individual or collective resource is still discussed. Kawachi, Subramanian and Kim (2008) defined social capital as an individual and collective attribute. In this logic, social capital resides in social structures, such as neighbourhoods and work environments, as well as in the sources that people access through their social networks (9).

Aiming to organize the different dimensions and levels of social capital, Rostila (2011) developed a theoretical model that includes the multiple faces of social capital (4). In this model, social capital comprises three fundamental components: social networks, social trust, and social resources. However, the first two components—social networks and trust—are considered preconditions for forming the third—social resources. Thus, social capital can be defined as the social resources that evolve from accessible social networks or social structures characterized by mutual trust, which facilitate access to different returns that can benefit the individual and communities (4).

Due to this exposed complexity around its construct, there is still no unmitigated method to measure social capital. Moreover, one might argue that social capital cannot be measured directly, but it can be inferred from its determinants or manifestations (1–4). The determinants are factors that influence social interactions, which allows social capital formation. On the other hand, manifestations are the results of social capital, such as perceived social support. Therefore, social capital is commonly measured through indicators or “proxies”, which are theoretically linked to the concept, both at an individual and community level (4).

Commonly used individual indicators of social capital refer to social participation, such as participation in organizations, political action and civic involvement (10, 11); social support and social networks, such as contact with friends and family, support systems, participation in religious activities and the depth of the relationships (12–14); and social trust, referring to the levels of trust and reciprocity in society and institutions in general (10). Community-level social capital has also been assessed. Some studies used indicators of community social networks, such as the number of formal entities in the community (association of workers, volunteers and community cultural centres) (13, 15, 16). Community social capital has also been evaluated through the number of participants in administrative assemblies, the number of homicides and volunteering (17, 18). Some questionnaires have also been developed to measure social capital or its dimensions (19).

The above-mentioned controversies suggest that there is a divergence and numerous ways to measure social capital. For this reason, most researchers remain using indicators, which generates criticism, since social capital is a multidimensional concept that is hardly completely measured with few variables. However, the use of indicators may be suitable in terms of public health, since it highlights the exact points of action for promoting health. In this context, social capital has been explored in different ways within health research.

## Social capital and health

The concept of social capital has been widely inserted in health discussions in recent decades (20). The “paradigm shifts” in health has suggested that the social environment has a significant effect on individuals’ health (21, 22). Social capital has become a popular term highlighted as one of the main determinants of health in the conceptual framework proposed by the World Health Organization (WHO) (23). Despite controversies and criticisms regarding its definition (24), a growing body of evidence suggests that high levels of social capital benefit health (20, 25).

Previous studies suggested that high levels of social capital are related to lower mortality rates, lower occurrence of chronic diseases, as well as lower rates of depression and suicide (26–29). It has also been demonstrated that high levels of social capital are associated with better mental health, better self-perception of health and better quality of life (27, 30). In addition, different aspects of health, including oral health, have also been explored in social capital literature.

## Social capital and oral health

Social capital has been associated with different normative and subjective oral health outcomes. Since different oral diseases are still considered a public health problem worldwide (31, 32), the factors that lead to the decline of oral health still need to be explored. Furthermore, currently and based on the contemporary definitions of health (33), oral health emerges as a positive concept that highlights the personal and social resources available to individuals, such as social capital.

Some studies evaluated the association between social capital and dental caries. Most studies showed that the higher level of individual social capital, the lower dental caries experience in children (34), adolescents (35), and adults (36). The same pattern has been observed regarding community social capital (37), where dental caries rates were significantly lower in areas with higher levels of empowerment. A 10-year follow-up cohort study also demonstrated that the high level of community social capital in early childhood directly impacted the lower incidence of dental caries in adolescence (38). This association was also mediated by behavioural and psychosocial variables (38).

Considering periodontal conditions, previous studies showed that high levels of social capital at the individual and community levels are related to less gingival bleeding in cross-sectional and longitudinal studies (14, 39, 40). Furthermore, a cross-sectional study involving Israeli adults demonstrated that religiosity led to greater social support through spirituality, which was related to lower levels of periodontitis (41).

A cross-sectional study performed on adults showed that low social capital was also associated with lower masticatory capacity (42). There is also evidenced on the positive association between individual- and community-level social capital and the number of natural teeth (43, 44). In addition, greater tooth loss over time (45) and more edentulism (46) have been related to lower levels of social capital.

Considering subjective outcomes, some previous studies evaluated the association between social capital and self-rated oral health (SROH), showing that the higher level of individual social capital, the better SROH in children (47), adolescents (48), and adults (45, 46). The same pattern has been observed regarding community social capital (47, 49), where SROH was significantly better in areas with higher levels of social capital. However, the social capital measured through high informal social control was related to poorer SROH in adolescents (49).

A positive relationship was also observed between high levels of social capital and oral health-related quality of life (OHRQoL). Previous literature showed that higher levels of social capital at individual and neighbourhood levels positively impacted on children's OHRQoL in cross-sectional and longitudinal studies (16, 38, 50). A positive association between high levels of social support and OHRQoL was also reported in adults (46). Furthermore, some studies have indicated an indirect influence of social capital on OHRQoL *via* dental status (48), stress (51) and sense of coherence (SOC) (38).

A recently systematic review evaluated the relationship between social capital and oral health in children and adolescents through 21 studies meta-analysed, totalling 81,241 individuals (52). It was demonstrated that community and individual social capital were positively related to oral health outcomes, such as dental caries, gingival bleeding, SROH and OHRQoL in children and adolescents. In general, community level social capital exerted more impact on oral health that individual social capital, and the subjective oral health outcomes were more affected (52). Systematic reviews with pooled measures of social capital and oral health in adults and the elderly have not been explored yet.

In this context, research on social capital and oral health have been conducted over the last two decades. However, in addition to exploring the association between social capital and oral health, testing interventions focused on oral health promotion are also relevant in terms of public health.

## Social capital and oral health promotion

Intervention studies focusing on health promotion through social capital remain scarce in previous literature. Considering general health, some intervention studies to promote social capital have been proposed (53–58).

A pragmatic randomized clinical trial involving older adults proposing a complex intervention to promote social capital, self-management, and health literacy showed positive effects on mental health and health promotion (55). Furthermore, the local governments and researchers in the Japan Gerontological Evaluation Study (JAGES) have conducted a community-based intervention program to enhance social capital and health. This intervention introduced community salons that proposed several activities to promote social networks and social participation. This program reduced the risk of poor self-rated health (SRH) (57) and cognitive decline (58).

A previous systematic review showed that social capital interventions improved mixed effects on general quality of life, well-being and SRH (53). In addition, another systematic review demonstrated that the majority of interventions in social capital are focused only on the individual level (54), not incorporating the environmental context where people are embedded. Finally, Flores et al., 2018 suggest the need for further high-quality trials, especially among vulnerable populations to assess the sustainability of the effect of social capital interventions (56).

To the best of our knowledge, there is no previous study assessing the effects of interventions on social capital and their impact on oral health promotion. However, a previous cluster-randomized trial demonstrated that a school-based intervention focusing on changes to enhance children's SOC improved their OHRQoL (59). In this sense, since previous studies have shown that SOC may interact with the coping and social capital of the individuals (60), interventions considering the interaction among these psychosocial factors seem promising to improve oral health.

## Future challenges and directions

Future research on social capital and oral health conditions has many challenges ahead. Additionally, different aspects of this relationship still need to be explored more deeply.

First, an important aspect to be considered is how social capital should be measured. The literature still lacks valid and standardized questionnaires that allow of global comparisons. Divergences in the conceptual of social capital and the consequent difficulties in measuring such complex construct may explain this methodological gap. Nevertheless, it is necessary to reflect on the paradigm shift about how friendship and social support networks are developed and sustained between people in the digital “era”. Thus, it is reasonable to rethink new possibilities for measuring social capital beyond traditional measures, also considering the interactions obtained through online social networks. Although some questionnaires have been suggested for this purpose (61), studies in oral health research that considers social capital in this way remained scarce in previous literature. Future studies considering social capital in digital environment may be important for planning future interventions and oral health promotion.

It is important to emphasize the scarcity of qualitative studies exploring the relationship between social capital and oral health outcomes. The use of mixed methods may be promising to enhance the knowledge in this topic, as well as for strengthening public health strategies to tackle social determinants. The use of these methods may also be useful for a better understanding of individuals’ perceptions of their social networks and social support. It should be noted that intervention studies involving community social capital are also scarce and are strongly encouraged, especially in oral health. Mapping mobilization of social capital in neighbourhoods may be one way of achieving community action for health promotion.

Another important factor to consider is the impact of the COVID-19 pandemic scenario on the social capital sources and on perceived social trust, which may impact individuals’ oral health. Previous studies have investigated social capital in a post-disaster context (62). Thus, this aspect still needs to be further explored in oral health promotion. Some studies have reported the role of social capital as a mediator between adverse conditions, such as low income and negative life events, and health outcomes (63). Moreover, further investigation of how social capital may interact with other factors and consequently impact oral health is also necessary.

Considering the current scenario, further research is needed to provide more in-depth knowledge on how social capital may affect oral health outcomes. Future challenges and directions in this area are specially related to the development of interventions and health promotion actions to stimulate social capital, aiming to reduce the impact of social inequalities on oral health throughout the life course.

## Data Availability

The original contributions presented in the study are included in the article/Supplementary Material, further inquiries can be directed to the corresponding author/s.
